# Does a relation between bone histomorphometry and fractures exist? The case of the equine radius and tibia

**DOI:** 10.17221/18/2024-VETMED

**Published:** 2024-09-26

**Authors:** Marco Zedda, Ramona Babosova, Sergio Gadau, Gianluca Lepore, Sara Succu, Vittorio Farina

**Affiliations:** ^1^Department of Veterinary Medicine, University of Sassari, Sassari, Italy; ^2^Department of Zoology and Anthropology, Constantine the Philosopher University in Nitra, Nitra, Slovak Republic

**Keywords:** bone tissue, Haversian canals, horse, osteons, traumatic events

## Abstract

Fractures of long bones in limbs are rare traumatic events in horses. This study investigates whether the incidence and types of fractures can be related to the histomorphometric features of the radius and tibia, which experience different biomechanical stresses and exhibit varying incidences and types of fractures. Clinical observations suggest that, in adults, slightly transverse and comminuted fractures are present in the radius, while the tibia shows a higher frequency of longitudinal and spiral fractures. Microscopic observations reveal no apparent distinctive characteristics between the radius and tibia, whereas the histomorphometric data highlight differences in the osteon density, eccentricity, and diameters of the osteons and Haversian canals. To sum up, tibial osteons are more numerous and smaller than those in the radius, resulting in a 15% higher total extension of the cement line in the tibia compared to the radius. These histomorphometric differences are an evolutionary adaptation to the different biomechanical stresses that involve the thoracic and pelvic limbs. Our results could help better understand numerous clinical realities detectable through retrospective analyses and aid in evaluating a specific bone’s predisposition towards traumatic events in all mammals, including humans.

Horse skeletons have evolved to enable these large animals to run at high speeds, and humans have taken advantage of this quality for an increasing number of events that expose the animals to traumatic injuries such as bone fractures. Equine fractures are relatively rare due to falls or collisions with external hard objects during races, whereas a kick from another horse frequently causes them (Sanders-Shamis et al. 1986; Auer 1999; Derungs et al. 2004; Theoret and Schumacker 2017) and, overall, by fatigue in racing horses, in which case, these internal fractures are termed fatigue fractures (Catcott and Smithcors 1975; Henry 2016; Nixon 2020; Ortved and Richardson 2021; Baxter 2022). In a retrospective study of more than one thousand external equine fractures, 47.2% resulted from a kick from another equid; among these, 85.6% involved bones of the limbs (Donati et al. 2018). Equine fractures do not affect all bones equally. The most frequently fractured bones are the second and fourth metapodial (metacarpal and metatarsal) bones, followed by skull bones, the first phalanx, the distal phalanx, and the pelvis (Donati et al. 2018).

It is also known that many types of bone fractures can be classified based on different criteria, such as the involvement of soft tissues with the skin surrounding the bone (open, closed fractures), the preservation of the bone axis (compound, displaced fractures), the extension of the fracture line (complete, fissure fractures), the presence of many bony splinters (comminuted fractures), and the direction of the break line in the bone (transverse, oblique, longitudinal, spiral fractures). More precisely, when the direction of the fracture line runs perpendicular to the long axis of the long bone, there is a transverse fracture; when the fracture line runs at an angle equal to or less than 45 degrees to the bone axis, there is a long oblique fracture; when the fracture line is greater than 45 degrees, there is a short oblique fracture; and when in oblique fractures the fracture line wraps around the long axis of the bone, there are spiral fractures, that are usually associated with torsional trauma (Henry 2016).

The equine bone microstructure has been deeply analysed to study its biomechanical properties and for comparative descriptions (Cuijpers 2006; Zedda et al. 2015; Pinilla et al. 2017), but a link between the incidence and type of fractures still needs to be considered (Currey 2005).

This work aims to describe the histomorphometric characteristics of the equine radius and tibia and verify if their possible structural differences could be related to the different incidence and kinds of fractures. The radius and tibia have been chosen for different reasons: 1) they belong to the same segment of the limb (zeugopodium); 2) during locomotion, they are involved in different biomechanical stresses; 3) their fractures show different incidences and types.

The radius and tibia in adult horses are extremely strong bones, and their fractures are associated with marked lameness, which has serious consequences (Sanders-Shamis et al. 1986). In a retrospective analysis of 17 horses with bone fractures, the authors reported that the radius was fractured in 9.1% of all forelimbs and the tibia in 10.0% of all hindlimbs fractured (Derungs et al. 2004). Other authors, conversely, indicate that, compared to other bones, fractures of the tibia are uncommon in horses, whereas fractures of the radius most commonly occur (Auer 1999; Schroeder et al. 2013). In a retrospective analysis including 1 144 horses with bone fractures, the incidence of fractures was 7.3% in the tibia and 6.8% in the radius (Donati et al. 2018). Unfortunately, in the clinical data reported in the literature, there is very often little or no information on the classification of the fractures identified. Furthermore, it must be added that the rankings and data reported in the references are often inconsistent due to the fact that the age of the animals is sometimes not taken into account. It is known that, in general, the long bones of foals are more frequently exposed to transverse fractures that coincide with the metaphyseal lines that are not yet ossified. This is due to the fact that the metaphyseal line is made up of cartilaginous tissue and represents the least robust part of the bone and therefore the most exposed to trauma. In this work, we are only taking adults into consideration, which, having completely ossified bones, are a good study model for looking for the relationship between the microstructure and the trend of the fracture lines.

As regards to adult horses, some different types of bone fractures have been observed in the radius, Auer (1999) describes fractures as spiraliform in 80% of cases and transversal in only 20% of cases. In contrast, Sanders-Shamis et al. (1986) affirm that the most common fractures are comminuted in 65% of cases and oblique in 35%. It is interesting to note that in the tibia of adult horses, there are no case reports of transversal fractures, and the only descriptions in the literature refer to diaphyseal, comminuted, oblique and spiraliform fractures (Rose and Hogson 2000; Schroeder et al. 2013).

Studies like this one, aimed at correlating the bone microstructure with the types of fractures, can pave the way to better understand the numerous clinical realities detectable by retrospective analyses and help evaluate the predisposition of a specific bone towards traumatic events in all mammals, including humans.

## MATERIAL AND METHODS

### Bone sampling and preparation

The material examined in this study consisted of 8 radii and 9 tibiae from horses (*Equus caballus*). All the bones belong to the osteological collection of the Department of Veterinary Medicine of the University of Sassari (Italy). The animals were adults and were regularly butchered in local slaughterhouses following a medical examination that ascertained their good health. The animals were not racehorses, and no evidence of skeletal lesions was detected in both types of bone.

### Histomorphometrical analyses

Sections from the smallest width of the diaphyses were observed and photographed using a Zeiss Axiophot microscope at × 2.5, × 10, and × 2.0 magnifications. In all sections, 247 secondary osteons were examined (132 in the sections of the radius and 115 in sections of the tibia). Only the intact secondary osteons were considered, and the following measurements were taken from the digitised images using the Scion Image software (Scion Corporation, Frederick, MD, USA): area, perimeter, minimum, and maximum diameters of the Haversian canals and secondary osteons. The eccentricity of the Haversian canals and secondary osteons was also calculated, and the osteon density and the extension and ratio between the area of secondary osteons and that occupied by the extraosteonal bone were evaluated.

### Statistical analyses

Statistical analyses were conducted using the NCSS software program. Pearson’s correlation coefficient between the size of the osteons and the Haversian canals was calculated, and the linear regression was drawn in a regression plotter. The variances within the size of osteons and Haversian canals were analysed for statistical significance (*P* < 0.05) using a one-way analysis of variance (ANOVA) with a Bonferroni post-hoc test.

## RESULTS

**Figure 1:**
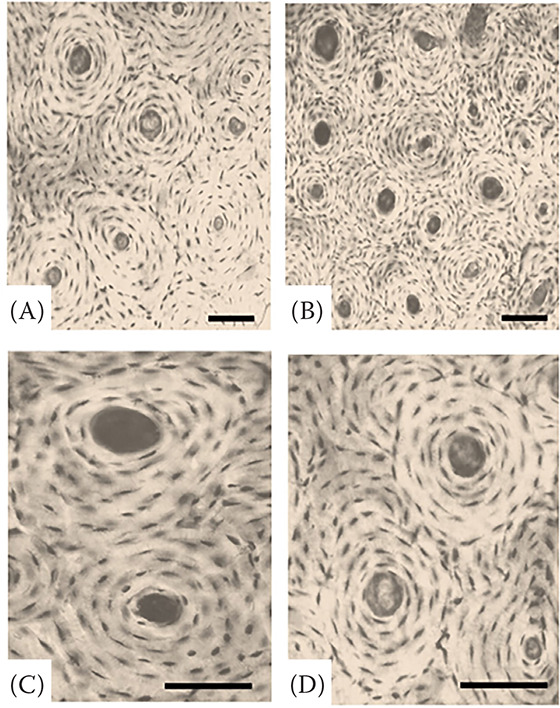
Horse bone sections: radius (A,C), tibia (B,D)

The general pattern of the bone tissue in both the radius and tibia is typical of the Haversian tissue (Figure 1), showing areas with a high number of secondary osteons longitudinally oriented and clustered together in small homogeneous groups (dense Haversian tissue).

Typical dense Haversian tissue is characterised by numerous clustered secondary osteons. Osteons consist of 6 to 10 bone lamellae surrounding the Haversian canal and are slightly elliptical. Transmitted light microscope. Bar** = **60 μm

Other osteons are more distant from each other and have a less precise course (irregular Haversian tissue). The extraosteonal bone matrix (Ex.BM) is here considered the area with no intact osteons. Then it also comprises the old osteons being demolished during the remodelling bone process, which appears scattered. This area, including both the subperiosteal and subendosteal zones, has an extension of 87.9% in the tibia and 89.4% in the radius (Table 1).

**Table 1:** Differences in histometric data between radius and tibia compared to the frequency and type of fractures. The values of the diameters and perimeters are expressed in μm and area in μm2

	Structures	Parameter acronym	Description	Radius	Tibia
Histometric data	bone sections	On.Dn	osteon population density	4.72	5.61
		Ex.BM	extraosteonal bone matrix	89.4%	87.9%
	osteons	maxOn.Dm	maximum diameter	179 ± 17	173 ± 16
		minOn.Dm	minimum diameter	166 ± 15	154 ± 13
		On.Dm	mean diameter	172 ± 16	165 ± 14
		On.Pm	perimeter	539 ± 62	516 ± 59
		On.Ar	area	23 213 ± 2 170	21 340 ± 1 925
		e	eccentricity	0.57	0.61
	Haversian canals	maxHC.Dm	maximum diameter	42 ± 3	38 ± 3
		minHC.Dm	minimum diameter	36 ± 7	30 ± 4
		HC.Dm	mean diameter	38 ± 3	33 ± 3
		HC.Pm	perimeter	118 ± 12	104 ± 11
		HC.Ar	area	1 133 ± 128	854 ± 83
		e	eccentricity	0.41	0.46
Clinical reports	frequency of slightly transversal fractures			+++	+
	frequency of spiraliform and oblique fractures			++	++++

Data referring to the clinical reports come from a synthesis of hundreds of descriptions of fractures reported in the references: (+) = truly rare occurrence; (++) = rare occurrence; (+++) = common occurrence; (++++) = frequent occurrence

The osteon density (On.Dn) differs between the radius (4.72 ± 2) and tibia (5.61 ± 3). Each osteon consists of 6 to 10 bone lamellae and is slightly elliptical in shape.

**Figure 2:**
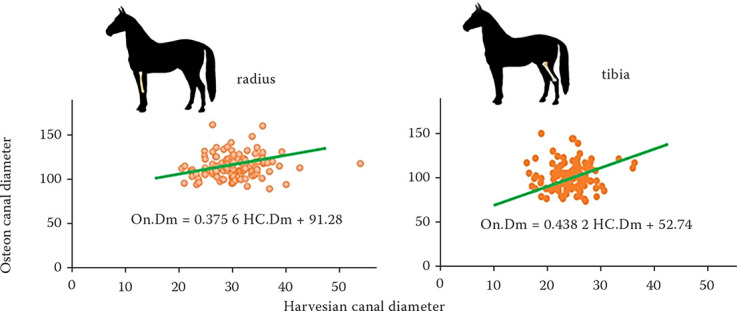
Scatter plots showing the correlation between the osteon diameter (On.Dm) and the Haversian canal diameter (HC.Dm) both in the radius and tibia

The osteons of the tibia (e** = **0.61) are slightly more elliptical than those of the radius (e** = **0.57). As regards the osteon size, all the parameters, such as osteon diameter (On.Dm), osteon perimeter (On.Pm), and osteon area (On.Ar), are slightly bigger in the radius than in the tibia. A similar trend in the differences between the radius and tibia can be noted in the Haversian canals, where the diameter (HC.Dm), perimeter (HC.Pm), and area (HC.Ar), continue to be slightly bigger in the radius than in the tibia. Regarding the eccentricity, the Haversian canals are slightly more circular in shape than their osteons, both in the radius and tibia (e** = **0.41 and e** = **0.46, respectively). The comparison of the size of the osteons (On.Dm) and their Haversian canals (HC.Dm) indicates a low positive correlation in the radius (Pearson’s correlation coefficient *r*** = **0.347) and a moderate positive correlation in the tibia (*r*** = **0.516), as reported in Table 2 and shown in Figure 2, where the regression lines were drawn in linear plotters, and their equations reported. The extension of the cement line, referred to as 1 square mm, is 2 540 μm in the radius and 2 930 μm in the tibia, showing a difference of about 15% between the bones in favour of the tibia.

**Table 2:** Statistical data on the variance of the osteon diameter, Pearson’s correlation, and regression line functions of the correlation between the diameter of the osteons and the Haversian canals

		Haversian canal diameter (HC.Dm)			
		variance	Pearson’s coefficient	function of regression line	correlation
Osteon diameter	radius	179.17	*r* = 0.347	On.Dm = 0.375 6 HC.Dm + 91.28	*
(On.Dm)	tibia	333.71	*r* = 0.516	On.Dm = 0.438 2 HC.Dm + 52.74	**

Data refer to mean diameter; Correlation: *low positive; **moderate positive correlation

The functions of each regression line are also reported; Values are expressed in μm

## DISCUSSION

The histomorphological observations are consistent with those reported in the literature, as many authors described Haversian tissues in adult equine bones (Cuijpers 2006; Pinilla et al. 2017; Zedda et al. 2020). This type of bone tissue is considered the best at resisting biomechanical stress, attributing the orderly parallel arrangement of osteons as the main structural feature responsible for its strength (Zedda 2023). It is accepted that the osteons, being formed by bone lamellae containing collagen fibres parallelly oriented within each lamella, are considered to have higher resistance than the extraosteonal bone that, despite being more mineralised, appears more frequently involved in microcracks (O’Brien et al. 2005; Mullins et al. 2009).

The very small difference (1.5%) detected in our study between the extension of the external bone matrix (Ex.BM) in the radius and tibia appears too negligible to justify the differences concerning the frequency and type of fractures.

The different osteon population density (On.Dn), higher in the tibia (5.61) than in the radius (4.72), and the different sizes of osteons could be a useful key to interpreting the data. A higher osteon population density would be correlated with higher exposure to mechanical stress, especially when associated with a reduction in the osteon size (Giua et al. 2014; Zedda et al. 2019; Zedda and Babosova 2021; Chiang and Xiaohua 2022).

As regards the shape of the osteons in the transversal section, they are elliptical, which is the best arrangement to support mechanical stresses, as observed in the long bones of other mammalian species (Palombo and Zedda 2016; Babosova et al. 2022; Palombo and Zedda 2022). The eccentricity is higher in the tibia (e = 0.61) than in the radius (e = 0.57), suggesting that the tibia, thanks to its structural morphology, is well-adapted to resist the great mechanical stresses, typical of the pelvic limb.

These remarks align with the understanding that the radius and tibia have different biomechanical roles during locomotion. The hindlimb bones are exposed to extremely considerable forces, bearing a large part of the body weight and initiating the thrust for movement. Conversely, the forelimb bones perform their main task of supporting part of the body weight and discharging the forces towards the ground during locomotion. It is worth noting that, in equine long bones, transverse fractures are associated with tension stresses, whereas oblique fractures occur during compression loads (Lopez 2019). This observation aligns with the different biomechanical stress to which the thoracic and pelvic limbs are subjected (Denoix 2015).

In recent years, there has been growing interest in the cement line as it is considered to be involved in the propagation dynamics of the fracture line. Its chemical composition is still a matter of debate, even though it is ascertained that it represents a hypermineralised structure with few collagen fibres. Cement lines are a weak interface between the osteons and the extraosteonal bone that absorb the mechanical energy during fractures, arresting short microfractures (Mullins et al. 2009; Chiang and Xiaohua 2022) and preserving the osteons. Since, in the tibia, the extension of the cement lines has been calculated to be 15% bigger than in the radius, and this could mean that, in the tibia, a major defence of the osteons occurs, favouring oblique and spiraliform fractures.

## Limitations of this work

The collection of data from the horses which the examined bones belonged to, represents one of the major limitations of this work. Indeed, the departmental osteological collection has grown over time thanks to donations of students and professionals without tracking the lifestyle of the horses to which the examined bones and the biomechanical stresses of their locomotion. However, the bones examined are a statistically significant sample that will pave the way for studies in this field.

Our study reveals that, in the tibia, osteons are smaller, but more numerous, whereas, in the radius, they are slightly larger but fewer in number. Therefore, slightly transverse fractures prevail in the radius, probably due to the fewer osteons. Conversely, a higher osteon population density in the tibia could be correlated with higher exposure to mechanical stress. The high frequency of longitudinal and spiral fractures in the tibia suggests that the fracture line would propagate more easily along the extraosteonal bone matrix, trying to dodge the osteons and resulting in a longitudinal split of the bone.

This work highlights how there are microstructural differences between the radius and tibia in the adult horses and these can certainly be attributed to the different biomechanical stresses suffered by the two bones during locomotion.

The question of predisposition to certain types of fractures rather than others remains open. A series of studies on all the other long bones and in horses with different locomotion conditions could help to resolve the question. If this is confirmed, histomorphometrical studies on bones could help predict the frequency and kind of bone fractures.
